# Effectiveness of passive ankle pump exercise on lower limb swelling in neonates with a peripherally inserted central catheter: a quasi-experimental study

**DOI:** 10.1590/1980-220X-REEUSP-2024-0275en

**Published:** 2025-04-14

**Authors:** Xiaoman Yu, Linlin Chen, Qian Wu, Wen Bi, Shanshan Yu

**Affiliations:** 1 The Affiliated Hospital of Qingdao University, Qingdao, China.; 2 Qingdao Eighth People's Hospital, Qingdao, China.

**Keywords:** Catheterization, Peripheral, Exercise, Cateterismo Periférico, Exercício Físico, Cateterismo Periférico, Ejercicio Físico

## Abstract

**Objective:**

To explore whether passive ankle pump exercises can relieve swelling in the lower limbs of newborns with a peripherally inserted central catheter.

**Method:**

A total of 129 neonates hospitalized in the neonatal intensive care unit of a tertiary hospital in Qingdao, from January to December 2023, were selected as the study subjects. They were divided into the control group (n = 64) and the intervention group (n = 65) by a convenient sampling method. The control group received routine intervention, while the intervention group was submitted to passive ankle pump exercise in addition to the routine intervention.

**Results:**

The changes in leg circumference over time in the two groups of newborns showed inconsistent trends (F = 93.99, p = 0.001). The passive ankle pump exercise reduced the increase in leg circumference by 17% in the intervention group. The median time for swelling resolution in the catheter limb of the intervention group was shorter than that of the control group (56 hours vs 80 hours), with a statistically significant difference in swelling resolution time between the two groups (F = 93.99, p = 0.001).

**Conclusion:**

The use of passive ankle pump exercise can reduce the degree of swelling in the lower limbs of neonates with a peripherally inserted central catheter and promote the resolution of swelling.

## RESUMO

**Objetivo:**

Explorar se os exercícios passivos de bombeamento do tornozelo podem aliviar o inchaço nos membros inferiores de recém-nascidos com um cateter central de inserção periférica.

**Método:**

Um total de 129 recém-nascidos hospitalizados na unidade de terapia intensiva neonatal de um hospital terciário em Qingdao, de janeiro a dezembro de 2023, foram selecionados como os sujeitos do estudo. Eles foram divididos em um grupo de controle (n = 64) e um grupo de intervenção (n = 65) por meio de um método de amostragem por conveniência. O grupo de controle recebeu uma intervenção de rotina, enquanto o grupo de intervenção foi submetido a exercícios passivos de bombeamento do tornozelo, além da intervenção de rotina.

**Resultados:**

As alterações na circunferência da perna ao longo do tempo nos dois grupos de recém-nascidos apresentaram tendências inconsistentes (F = 93,99, p = 0,001). O exercício passivo de bombeamento do tornozelo reduziu o aumento da circunferência da perna em 17% no grupo de intervenção. O tempo médio para a resolução do inchaço no membro do cateter do grupo de intervenção foi menor do que o do grupo de controle (56 horas vs. 80 horas), com uma diferença estatisticamente significativa no tempo de resolução do inchaço entre os dois grupos (F = 93,99, p = 0,001).

**Conclusão:**

O uso de exercícios passivos de bombeamento do tornozelo pode reduzir o grau de inchaço nos membros inferiores de recém-nascidos com um cateter central de inserção periférica e promover a resolução do inchaço.

## RESUMEN

**Objetivo:**

Explorar si los ejercicios pasivos de bombeo del tobillo pueden aliviar la hinchazón en las extremidades inferiores de los recién nacidos con un catéter central de inserción periférica.

**Método:**

Se seleccionaron como sujetos del estudio 129 recién nacidos hospitalizados en la unidad de cuidados intensivos neonatales de un hospital terciario de Qingdao de enero a diciembre de 2023. Se dividieron en un grupo de control (n = 64) y un grupo de intervención (n = 65) mediante un método de muestreo de conveniencia. El grupo de control recibió una intervención rutinaria, mientras que el grupo de intervención se sometió a ejercicios pasivos de bombeo del tobillo además de la intervención rutinaria.

**Resultados:**

Los cambios en la circunferencia de la pierna a lo largo del tiempo en los dos grupos de recién nacidos mostraron tendencias inconsistentes (F = 93,99, p = 0,001). El ejercicio pasivo de bombeo del tobillo disminuyó el aumento de la circunferencia de la pierna en un 17% en el grupo de intervención. El tiempo medio de resolución de la hinchazón en la extremidad con catéter del grupo de intervención fue más corto que el del grupo de control (56 horas frente a 80 horas), con una diferencia estadísticamente significativa en el tiempo de resolución de la hinchazón entre los dos grupos (F = 93,99, p = 0,001).

**Conclusión:**

El uso de ejercicios pasivos de bombeo del tobillo puede reducir el grado de hinchazón en las extremidades inferiores de los recién nacidos con un catéter central de inserción periférica y favorecer la resolución de la hinchazón.

## INTRODUCTION

Peripherally inserted central catheters (PICCs) offer advantages such as avoiding frequent needle punctures and reducing vascular damage from irritating medications^(1,2)^, and are widely used in Neonatal Intensive Care Units (NICU). Due to the higher success rate of single-attempt catheterization and the lower overall complication rate compared to upper limb vein catheterization, related guidelines recommend prioritizing the use of lower limb veins for neonatal PICC insertion^(3,4)^.

The NICU of the Affiliated Hospital of Qingdao University conducts clinical practice in accordance with guideline requirements and has observed a higher incidence of lower limb swelling on the catheterized side in neonates. Literature indicates that limb swelling is often associated with catheter-related thrombosis^(5,6)^. After limb swelling occurs, an ultrasound examination is first conducted to rule out thrombosis. Once catheter-related thrombosis is ruled out, the swelling appears to be attributed to factors specific to neonates, such as the narrowness of their blood vessels, which can impede circulation after catheter insertion^(7)^, and because infusion devices restrict limb movement when a PICC is inserted in the lower limb. Mild cases of swelling may affect catheter usage and cause limb discomfort in neonates, while severe cases may require catheter removal. For the prevention of limb swelling, the guidelines recommend that catheter diameter should be less than one-third of the vessel diameter^(8)^. However, in China, only 1.9Fr PICCs are currently available for the neonatal population, limiting clinical practice to this specific catheter size. The guidelines do not directly provide specific measures for managing swelling limbs; thus, in clinical nursing practice, neonates are often positioned with the affected limb elevated, and gentle warm compresses are applied with caution to allow the swelling to subside gradually.

Studies in adults have demonstrated that different modes of ankle pump exercises can prevent PICC-related thrombosis through the femoral vein^(9)^. Quantified grip exercises have been shown to effectively reduce the incidence of PICC-related thrombosis and infection and improve venous hemodynamics. The “2021 Infusion Therapy Standards of Practice”^(10)^ encourages patients with indwelling PICCs to engage in early, routine daily activities and mild physical exercise to promote blood flow.

The ankle pump exercise centers on the ankle joint, simulating the calf muscle pump activity during normal walking. Through dorsiflexion and plantarflexion of the ankle, it engages the lower limb muscle groups to promote contraction and relaxation, thereby enhancing the calf muscle pump function. The exercise involves rhythmic contraction and relaxation of the soleus and tibialis anterior muscles, which serve as a pump to accelerate venous blood flow in the lower limbs^(11)^. Since neonates cannot move their limbs voluntarily according to instructions, this study applied ankle pump exercises to relieve lower limb swelling on the catheterized side in neonates. A designated nurse performed passive movements on the infants, and the results of this intervention are reported below.

## METHOD

Convenience sampling was used to select 129 neonates with a PICC through lower limb vein who were admitted to the Affiliated Hospital of Qingdao University, from January to December 2023, as the intervention and control groups. Inclusion criteria were: (1) Neonates with PICCs placed in the lower limbs according to the PICC catheterization norms; (2) PICC catheter tip located in the inferior vena cava; and (3) Neonates whose parents provided informed consent. Exclusion criteria were: (1) Neonates with pre-existing lower limb swelling at the time of catheterization; and (2) Neonates with lower limb swelling due to disease. Dropout criteria were: (1) Neonates who were transferred out of the NICU or died during the study period; (2) Neonates who developed deep vein thrombosis in the lower limbs during the intervention; and (3) Neonates with unplanned catheter removal during the study.

The study was reported based on the CONSORT Statement for Randomized Trials of Nonpharmacologic Treatments^(12)^. This research proposal was approved by the Ethics Committee of the Affiliated Hospital of Qingdao University.

A nurse certified in PICC placement performed the catheter insertion using a 1.9Fr catheter, with ultrasound guidance for venipuncture and bedside ultrasound for tip positioning. When swelling appeared in the catheterized lower limb of a neonate, an ultrasound consultation was first performed to check for deep vein thrombosis. If detected, thrombolytic and anticoagulant treatments were administered, and the study was discontinued for that neonate.

*Control group*: After catheterization, neonates were placed in a natural position. No special treatment was given within 24 hours after catheter placement to prevent bleeding at the puncture site. After 24 hours, the limb with the PICC was elevated 20-30°, and warm compresses were applied to keep them warm.

**Figure 1:**
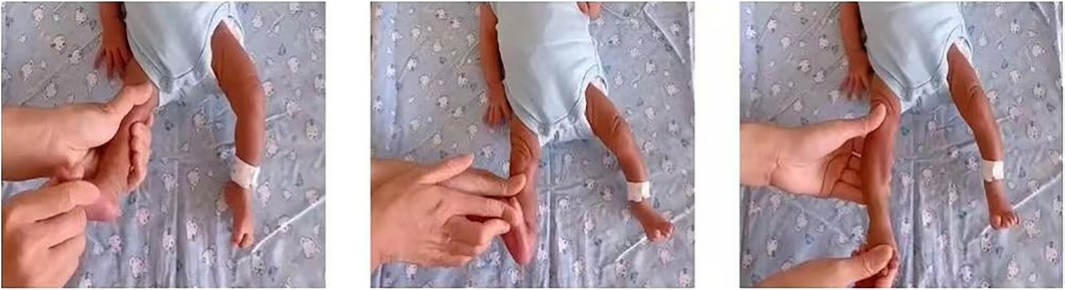
Passive ankle pump exercise. Qingdao, Shandong, China, 2023.

*Intervention group*: In addition to the same measures as the control group, passive ankle pump exercises were performed three times daily for 10 minutes each time. The exercise involved maximal dorsiflexion and plantar flexion of the foot for 5 seconds each, followed by 30 ankle rotations per minute for 5 minutes^(13,14)^. The specific operation method can be seen in Figure 1. The principle behind the ankle pump exercise accelerating blood flow is that during ankle joint movement the calf muscles contract and compress the intramuscular veins and deep veins, increasing venous pressure and pushing blood from the lower limbs toward the heart. As venous pressure rises, both the maximum venous outflow and maximum venous capacity increase, leading to an increase in blood flow velocity. The ankle pump exercise also exhibits a piston effect, where the propulsive pressure generated during dorsiflexion can be transmitted through the majority of the calf muscles into the fascial sheath, expelling blood into the distal veins^(15)^.

Maximal dorsiflexion Maximal plantar flexion Ankle rotations

*Evaluation indicators*: Limb swelling is determined by measuring the circumference of the leg. For neonates’ lower limbs with a PICC, the thigh circumference was measured. The measurement method was as follows: A measuring tape was wrapped around the midpoint of the line connecting the anterior superior iliac spine and the upper edge of the patella. The unit of measurement was centimeters, accurate to two decimal places. Immediately after the PICC insertion, the neonate’s leg circumference was measured. Subsequently, the circumference of both legs was monitored every 8 hours (once per shift) and compared with the skin on the non-catheterized side. Two people jointly determined and recorded the presence of limb swelling. For neonates who developed limb swelling, the time of onset and the time when the swelling subsided were recorded. Follow-up lasted for 7 days from the start of PICC insertion.

*Quality control*: Experienced and skilled nurses and infusion therapy nurses were incorporated into the research team, led by the head nurse. The head nurse provided training and assessments for team members on limb measurement and passive ankle pump exercises. To ensure consistency in data collection, the head nurse conducted weekly on-site evaluations to assess team members’ operational proficiency.

Strict adherence to inclusion and exclusion criteria was kept in selecting subjects. Unified PICC brand, model, fixation method, and dressing. Consistent personnel for catheter placement, dressing changes, and data collection.

*Data collection*: General information such as gestational age, weight, age, catheter placement site, and thigh circumference of the catheterized limb was collected. The occurrence and duration of limb swelling and catheter-related thrombosis were recorded.

*Statistical methods*: Data were analyzed using SPSS 26.0 and Excel 2020 software. Gender and catheter placement sites were described by frequency and analyzed by chi-square test. Gestational age and weight were described by mean ± standard deviation and analyzed by t-test. Changes in leg circumference were described as percentages to account for measurement site differences. Repeated measures ANOVA was used to analyze leg circumference changes between the two groups, with Greenhouse-Geisser correction applied. Kaplan-Meier method was used to draw survival curves for the resolution of swelling, and the log-rank test was used to compare differences between the two groups.

## RESULTS

### General Information

The study included 129 neonates (70 males, 59 females). The control group was removed for one newborn with catheter-related thrombosis. There were no significant differences in general information between the two groups (Table 1).

**Table 1:** Comparison of general data between the two neonatal groups – Qingdao, Shandong, China, 2023.

Index	Control group(64)	Intervention group(65)	χ^2^/t	p
**Gender**				
Male	34	36	0.066^1^	0.797
Female	30	29		
**Primary diagnosis**			1.914^3^	0.752
Extremely preterm infant	23	27		
Extremely low birth weight infant	19	15		
Very low birth weight infant	13	12		
NRDS	6	5		
Neonatal intestinal obstruction	3	6		
**Gestational age (week)**	31.95 ± 4.71	32.98 ± 4.87	1.22^2^	0.22
**Weight (kg)**	2.01 ± 0.22	2.00 ± 0.23	0.01^2^	0.99
**Catheter location**			1.75^1^	0.63
Great saphenous veins	41	35		
Small saphenous veins	4	7		
Popliteal vein	14	16		
Femoral vein	5	7		
**Number of punctures**			1.283^3^	0.562
1	17	13		
2	33	33		
3	14	19		
**Catheter-related complications**				
CRBSI	1	2	–0.567^3^	0.572
Phlebitis	12	15	–0.600^3^	0.549
Catheter-related thrombosis	1	0	1.024^3^	0.312

^1^Represents χ^2^

^2^Represents t

^3^Represents Fisher’s exact probability method. *Changes in leg circumference.*

**Figure 2:**
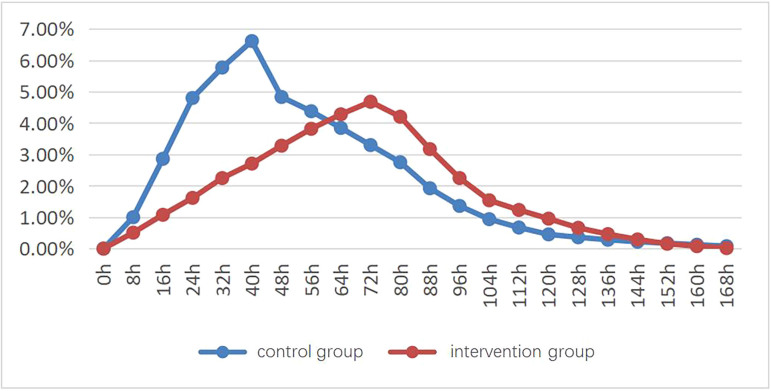
Trend of evolution of swelling in both groups – Qingdao, Shandong, China, 2023.

The leg circumference of the control group peaked at 40 hours post-catheterization with an average increase of 6.61%. The intervention group peaked at 72 hours with an average increase of 4.68%, indicating that passive ankle pump exercise delayed the peak and reduced the extent of swelling. By 144 hours post-catheterization, the leg circumference increase was similar in both groups (Figure 2).

The area under the curve for the control group was 0.47, while for the intervention group, it was 0.39, showing a 17% reduction in swelling extent due to passive ankle pump exercise.

### Repeated Measures Anova of Leg Circumference Changes

Mauchly’s test of sphericity showed non-sphericity (χ^2^ = 4056.84, P < 0.001), so Greenhouse-Geisser correction was applied (ε = 3.47). Results showed significant main effects of group (F = 4.66, P = 0.03) and time (F = 456.85, P < 0.001), and a significant interaction between group and time (F = 93.99, P < 0.001), indicating differences in leg circumference changes over time between the two groups (Table 2).

**Table 2:** Intra-group and between-group effect tests of changes in leg circumference in the two groups – Qingdao, Shandong, China, 2023.

	Class III and the sum of the squares	Free degree	Mean square	F	*P*
Intercept	1.08	1	1.08	611.05	0
Group	0.01	1	0.01	4.66	0.03
Time	0.77	3.47	0.221	456.85	0.001
Time*group	0.16	3.47	0.046	93.99	0.001

### Comparison of Swelling Resolution Time

In the control group, 38 neonates developed limb swelling, with 3 still swollen at the end of follow-up. In the intervention group, 29 neonates developed swelling, with 2 still swollen at the end of follow-up.

**Figure 3:**
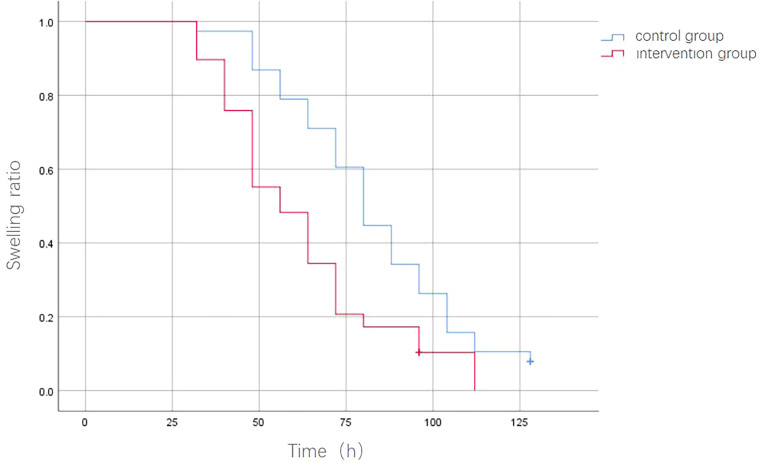
Kaplan-Meier curves of time to swelling resolution in both groups. Qingdao, Shandong, China, 2023.

Kaplan-Meier survival curves showed a median swelling resolution time of 80 hours for the control group and 56 hours for the intervention group, with a significant difference between the two groups (Tables 3 and 4, Figure 3).

**Table 3:** Median time to resolution of swelling (h) in both groups of neonates – Qingdao, Shandong, China, 2023.

Group	Median	Standard error	95% confidence interval	
			Lower limit	Upper limit
Control group	80.00	4.09	71.99	88.01
Intervention group	56.00	7.18	41.94	70.07
Total	72.00	4.06	64.05	79.95

**Table 4:** Comparison of resolution of limb swelling in the two groups – Qingdao, Shandong, China, 2023.

	χ^2^	Free degree	*P*
Log-Rank (Mantel-Cox)	8.56	1	0.001

## DISCUSSION

### High Incidence and Significant Impact of Limb Swelling in Neonates with Lower Extremity Picc

Limb swelling on the side of lower limb PICC catheterization in neonates is a common complication, yet there is a lack of effective preventive or intervention measures. The study showed that out of 129 neonates with a PICC in the lower extremity, 67 exhibited varying degrees of swelling, with an incidence rate of 51.94%. This high incidence indicates that limb swelling due to PICC placement is a significant issue. Comparisons with domestic and international studies reveal that there are few reports of limb swelling caused by PICCs, possibly due to the reasons explained below.

Many studies consider limb swelling as part of phlebitis. The INS guidelines indicate that stage 2 phlebitis involves pain at the infusion site with redness and/or edema. Since neonates cannot verbally express pain at the infusion site, edema is the most visible sign. Aiko’s^(16)^ study evaluated swelling and induration at the PICC insertion site through palpation, showing an awareness of swelling at the insertion site. This study reported a 34.5% incidence of phlebitis after palpation evaluation.

Recommendations for PICC insertion in the lower extremity of neonates have only emerged in the past two years^(17,18)^. Reports on complications related to lower extremity PICC insertion mainly focus on infectious, mechanical complications, and catheter-related thrombosis^(19,20)^. Comprehensive implementation in domestic neonatal intensive care units is still lacking, and some studies still recommend upper extremity PICC insertion for neonates^(21)^. Therefore, research on limb swelling is scarce.

The swelling observed after lower extremity PICC insertion in neonates is essentially limb edema, which is palpable swelling caused by an increased fluid volume in the interstitial space. It can be localized or systemic, with PICC-related limb swelling being localized. When swelling occurs in the catheterized limb, thrombosis should be ruled out first. In this study, swelling was mainly observed below the knee joint. The natural flexion position of the neonate’s lower limbs, combined with PICC insertion, reduces venous return, leading to swelling. Consultations with vascular surgeons and ultrasound specialists indicated that swelling in the catheterized limb was related to impaired venous return caused by the PICCs. Impeded blood return is a significant factor in venous thrombosis formation^(22)^. The incidence of PICC-related thrombosis in neonates is 9.2-13.2%^(23)^, with an incidence of 3.5-16% for central venous catheter thrombosis in the lower extremities^(24)^. Reports indicate that catheter-related thrombosis accounts for 89% and 94% of neonatal thrombosis cases in Canada and the Netherlands, respectively^(25)^. Therefore, effective measures should be sought to promote blood return in the catheterized limb to alleviate swelling. Current clinical interventions mainly include limb massage, elevation of the catheterized limb, and warm compresses. However, since neonates cannot maintain limb elevation independently and warm compresses pose certain risks, other effective measures should be actively sought.

### Ankle Pump Exercises Can Effectively Prevent Swelling and Promote Recovery in the Catheterized Limb

The study results show that in the control group, under routine care, the circumference of the catheterized limb peaked at 40 hours after puncture, while in the intervention group, with ankle pump exercises, the peak was delayed until 72 hours (lower than the peak in the control group). This indicates that ankle pump exercises delayed swelling in the catheterized limb to some extent, having a preventive effect. At 144 hours (6 days) after puncture, the swelling in both groups of neonates became similar, suggesting that swelling would naturally subside after 6 days even without ankle pump exercises, due to the body’s self-regulation.

Repeated measures ANOVA of limb circumference changes in both groups of neonates indicated that the changes over time were inconsistent between the groups. The median swelling resolution times were 80 hours and 56 hours for the two groups, respectively, with significant statistical differences. This shows that ankle pump exercises play an important role in promoting the resolution of limb swelling. This study adopted the exercise regimen from Lu Xiaomei et al.’s research^(7)^. The principle of ankle pump exercises^(26)^ involves active movements of the lower limb muscles (such as the quadriceps, tibialis anterior and posterior, soleus, gastrocnemius, peroneus longus and brevis) through dorsiflexion and plantar flexion of the ankle joint, promoting blood return by muscle contraction and improving peak venous blood flow and blood return speed.

Ankle pump exercises are a simple, practical, and cost-effective exercise method widely used in clinical adult patients. Studies show^(27,28,29)^ that ankle pump exercises can effectively prevent deep vein thrombosis in the lower extremities, promote lower limb blood circulation, and effectively alleviate lower limb swelling. However, clinical application standards for ankle pump exercises vary, and there are no reports on their use in neonates. This study proves that ankle pump exercises can effectively relieve swelling in the catheterized limb of neonates with lower extremity PICC and promote the recovery of swollen limbs. The simplicity and feasibility of this intervention make it highly applicable in clinical practice. Future studies should further explore the optimal duration and parameters of passive ankle pump exercise to achieve the best outcomes with minimal intervention.

## CONCLUSION

The results of this study indicate that the use of passive ankle pump exercises can reduce the degree of swelling in the lower limbs of neonates with PICCs and promote the resolution of swelling.
